# 环孢素A在反相液相色谱中的吸附行为及分离纯化

**DOI:** 10.3724/SP.J.1123.2021.01045

**Published:** 2022-01-08

**Authors:** Zhidong LI, Qing FU, Zhuoshun DAI, Yu JIN, Xinmiao LIANG

**Affiliations:** 1.华东理工大学药学院制药工程与过程化学教育部工程研究中心, 上海 200237; 1. Engineering Research Center of Pharmaceutical Engineering and Process Chemistry, Ministry of Education, School of Pharmacy, East China University of Science and Technology, Shanghai 200237, China; 2.中国科学院分离分析化学重点实验室, 中国科学院大连化学物理研究所, 辽宁 大连 116023; 2. CAS Key Laboratory of Separation Science for Analytical Chemistry, Dalian Institute of Chemical Physics, Chinese Academy of Sciences, Dalian 116023, China

**Keywords:** 反相液相色谱, 制备高效液相色谱, 环孢素A, 吸附等温线, 纯化, reversed-phase liquid chromatography (RPLC), preparative high performance liquid chromatography (prep-HPLC), cyclosporine A (CsA), adsorption isotherm, purification

## Abstract

环孢素A(cyclosporine A, CsA)是由11个氨基酸组成的中性环状多肽,是临床拮抗器官和组织移植后排异反应的首选药物。高效液相色谱法广泛应用于CsA的分离分析,开展CsA色谱行为的研究是使用制备高效液相色谱纯化CsA的关键。该文首先在C18固定相上比较了CsA在甲醇-水和乙腈-水两种流动相体系中的保留行为,结果表明其保留时间对有机相比例变化比较敏感。控制甲醇比例在84%~88%,或者乙腈比例在75%~85%, CsA的保留因子(*k*)在3~7范围内。考察了上样量对CsA峰形的影响。随着上样量增加,在两种流动相体系中,CsA的峰形都由对称开始变得拖尾,保留时间前移。因此在进行CsA纯化时,需要特别注意前杂的去除情况。然后采用吸附等温线描述CsA的保留行为,当流动相中CsA的质量浓度较低时,有机相比例对溶质在固定相上的吸附量影响并不明显。随着溶质的质量浓度增加至0.5 g/L以上,有机相比例降低有助于提高CsA在固定相上的吸附量。和甲醇-水体系相比,在乙腈-水体系中固定相对溶质有更大的吸附容量。用模型对CsA的等温吸附曲线拟合,在甲醇-水体系中符合Langmuir模型,在乙腈-水体系中为Moreau模型。由模型参数可知在两种体系下,CsA在C18固定相上均为单层吸附,区别在于乙腈-水体系中CsA会产生较大的分子间作用力。最后,实验采用0~60 min 65%~75%乙腈、60~80 min 75%乙腈的条件开展了环孢素A纯化的探索实验,可将CsA的杂质控制在0.2%以下。本研究结果对采用制备高效液相色谱纯化CsA具有指导意义。

## Abstract (en)

High performance liquid chromatography (HPLC) is widely used in the separation and analysis of cyclosporine A (CsA). Analyzing the chromatographic behavior of CsA is key to the purification of CsA by preparative HPLC. In this study, the retention behavior of CsA on the C18 column using mobile phases of methanol-water and acetonitrile-water was compared. The retention time of CsA was sensitive to the change in the ratio of the organic solvent. When 84%-88% methanol or 75%-85% acetonitrile was used, the retention factor (*k*) was in the range of 3-7. The change in the peak shape of CsA was investigated with loading amounts of 5, 25, 50, 100, and 500 mg. With an increase in sample loading, the peak shape of CsA in both mobile phases changed from symmetric to tailing, and the retention time reduced. Therefore, it is necessary to focus on the removal of impurities that were eluted before CsA during the purification. In addition, the peak shapes of CsA in methanol-water and acetonitrile-water were similar in the tested concentration range. This indicates that it was not possible to tune the peak shape of CsA by changing the organic solvent. Adsorption isotherms were obtained to describe the retention behavior of CsA. When the mass concentration of CsA in the mobile phase was low, the effect of the organic solvent ratio on the adsorption capacity of CsA on the C18 stationary phase was not distinct. With an increase in the solute mass concentration above 0.5 g/L, the reduced proportion of organic solvent helped improve the adsorption capacity of CsA. When the mass concentration of CsA in the mobile phase reached 5 g/L, the adsorption capacities were 24.9 g/L in 88% methanol and 40.8 g/L in 84% methanol. The adsorption capacity of CsA in acetonitrile-water was higher than that in methanol-water. When the mass concentration of CsA was 5 g/L, the adsorption capacity increased to 46.4 g/L in 75% acetonitrile. Scatchard analysis showed that the slope of the adsorption isotherm decreased gradually, which was consistent with trend observed in the Langmuir adsorption isotherm for the shape of the Langmuir peak (i.e. trailing peak). When the mass concentration of CsA in the mobile phase was between 0.01 g/L and 0.03 g/L, the slope of the curve decreased significantly, and the peak shape of CsA rapidly tailed with increasing loading amount. However, when using a mobile phase with a lower proportion of organic solvent (84% methanol or 75% acetonitrile), this trend was weakened. The adsorption data of CsA were fitted to models. The Langmuir model was found to be suitable for the methanol-water mobile phase, and the Moreau model for the acetonitrile-water mobile phase. The model parameters indicated that the monolayer adsorption of CsA occurred on the C18 stationary phase in both mobile phases, the difference being that more intermolecular interactions between CsA occurred in the acetonitrile-water mobile phase, resulting in a higher adsorption capacity. In methanol-water, the intermolecular interactions between CsA were inhibited by methanol due to its role as a proton donor. As an aprotic solvent, acetonitrile could only weakly inhibit these interactions; hence, the interactions could be improved by increasing the acetonitrile proportion. As the proportion of acetonitrile changed from 85% to 75%, the saturated adsorption capacity increased from 123 g/L to 197 g/L, while the interaction constant decreased from 0.618 to 0.588. Finally, CsA was purified using the conditions of 0-60 min 65%-75% acetonitrile, 60-80 min 75% acetonitrile, by which the impurity could be controlled to below 0.2%. The results of this study will aid in the purification of CsA by preparative HPLC.

环孢素A(cyclosporine A, CsA)是由11个氨基酸组成的中性环状多肽(见图1),在临床作为拮抗器官和组织移植后排异反应的首选药物^[1,2,3]^。在CsA的分离检测中,使用最广泛的是高效液相色谱法(HPLC)^[4,5]^。依据CsA的脂溶性特点,HPLC分析时多采用反相C18固定相,流动相以乙腈-水、甲醇-水为主,添加乙酸铵、甲酸、磷酸、三氟乙酸等添加剂^[6,7,8,9,10,11]^。有文献报道,CsA在HPLC中的保留时间随着温度升高没有明显降低。这是因为CsA具有独特的环状刚性结构,在高温下的水溶剂化能力较差,所以流动相的洗脱能力并没有随着升温而显著提高,CsA的保留时间波动很小^[12,13]^。

**图 1:**
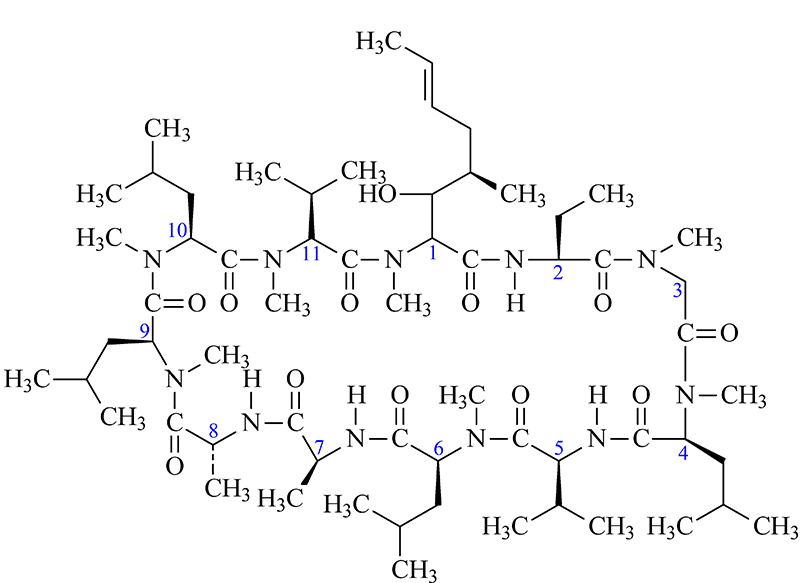
环孢素A的结构

已报道的CsA及其衍生物的纯化方法包括了大孔树脂、柱层析和高速逆流色谱等^[14,15,16]^,发酵液经过大孔树脂处理后,可以达到去除杂质和富集环孢素的目的,而随后采用硅胶柱层析或者高速逆流色谱法则可纯化得到环孢素单体。为了进一步去除杂质,达到杂质的限量要求,同时提高上样量、回收率等工艺参数,更多先进的分离技术用于药品的纯化制备。制备高效液相色谱(prep-HPLC)具有柱效高、分离快速稳定、在线检测和自动收集的优势,已广泛用于发酵来源的药物和天然产物这类复杂样品的分离纯化。开展CsA的色谱行为研究,深入了解CsA的色谱特性,可为CsA制备方法的选择与优化提供理论上的指导。从影响反相液相色谱(RPLC)制备的关键因素出发,本文系统开展了CsA在RPLC模式下的保留行为研究,包括考察有机溶剂种类和比例对CsA保留的影响,探索CsA峰形和上样量之间的关系。并通过测定CsA的吸附等温线,对其色谱行为给出理论上的支持。基于上述结果,尝试开展CsA的纯化分离,为prep-HPLC纯化CsA提供参考。

## 1 实验部分

### 1.1 仪器、试剂与材料

Waters Alliance高效液相色谱仪(Waters, USA),包括2695四元梯度泵、2489 UV-Vis检测器、自动进样器和柱恒温系统;XP105DR分析天平(Mettler Toledo, 瑞士)。环孢素A原料药(纯度>99%)和粗品为浙江瑞邦药业有限公司提供,分别用于吸附量测定和分离纯化实验。

色谱级甲醇(MeOH)、乙腈(ACN)、甲基叔丁基醚、磷酸(H_3_PO_4_,纯度>98%)购自百灵威(中国)科技有限公司;流动相水为娃哈哈纯净水,经0.22 μm膜过滤。

### 1.2 实验条件

1.2.1 流动相体系对环孢素A保留的影响

称取CsA原料药适量,用甲醇溶解,配制质量浓度为10 g/L的样品溶液,用0.22 μm膜过滤备用。C18色谱柱(150 mm×4.6 mm, 10 μm, 10 nm, 浙江华谱新创科技有限公司);在甲醇-水和乙腈-水体系下采用等度洗脱,甲醇-水的比例分别为80/20、82/18、84/16、85/15、86/14、88/12、90/10 (v/v),乙腈-水的比例分别为60/40、65/35、70/30、75/25、80/20、85/15、90/10(v/v)。进样量5 μL,流速1.0 mL/min;柱温55 ℃,检测波长203 nm。

1.2.2 考察上样量对环孢素A峰形的影响

分别称取适量CsA溶于甲醇中,配成质量浓度为1、5、10、20和40 g/L的样品溶液。流动相分别采用甲醇-水(88/12,86/14,84/16, v/v)及乙腈-水(85/15,80/20,75/25, v/v),其余条件同1.2.1。

1.2.3 测定吸附等温线

采用前沿吸附法测定吸附等温线^[17]^,以甲醇-水(84∶16)体系为例:(1)配制84%甲醇水溶液,过0.22 μm膜作为流动相A。(2)分别称取25、250、1250 mg CsA,充分溶解于A相溶液中,转移至250.00 mL容量瓶中,用A相溶液定容至刻度线,分别得到样品质量浓度为0.1、1、5 g/L的84%甲醇溶液,过0.22 μm膜,分别作为流动相B、C、D。

实验方法(以甲醇-水体系为例) 色谱柱:C18 (50 mm×4.6 mm, 10 μm, 10 nm, 浙江华谱新创科技有限公司),柱温:55 ℃;流速:1.0 mL/min;用A相充分平衡,开始进样,进样体积设置为0 μL。依次设置流动相A、B、C和D的比例,以台阶梯度的方式改变流动相中CsA的浓度。控制B(C或D)的体积分数为0、10%、20%、30%、40%、50%、60%、70%、80%、90%、100%,对应流动相中样品的质量浓度分别为0、0.01、0.02、0.03、0.04、0.04、0.05、0.06、0.07、0.08、0.09和0.1 g/L(C: 0.1~1 g/L, D: 0.5~5 g/L)。每个台阶梯度时间为10 min。为了使紫外检测在线性范围内,适当调整检测波长,对应B、C、D相的检测波长分别为230、245和255 nm,环孢素A的紫外光谱图见图2。在乙腈-水体系中的实验方法同上。

**图 2:**
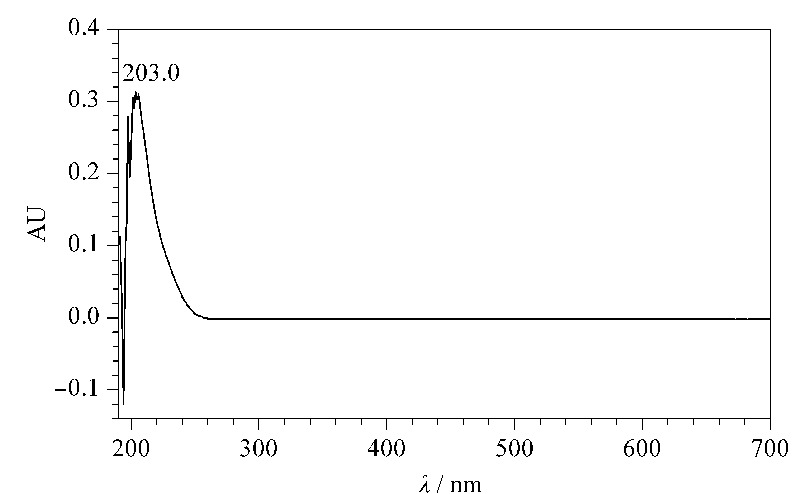
环孢素A的紫外光谱图

死时间*t*_0_的测定 称取KNO_3_适量,配制质量浓度为5 g/L KNO_3_溶液,过0.22 μm滤膜备用。分别在对应的流动相条件下进KNO_3_溶液,进样体积10 μL;洗脱方式:0~1 min, 100%A,记录KNO_3_出峰时间,重复3次,取平均,记为*t*_0_。

柱外流经时间*t*_1_的测定 在对应的流动相条件下,取下色谱柱,用两通连接两端管路,洗脱方式:0~0.5 min, 100%A; 0.5~5 min, 100%B。取曲线上升到一半高度时的保留时间减去A相流经的0.5 min即为柱外流经时间*t*_1_(示意图见图3a)。重复3次,取平均值。

**图 3:**
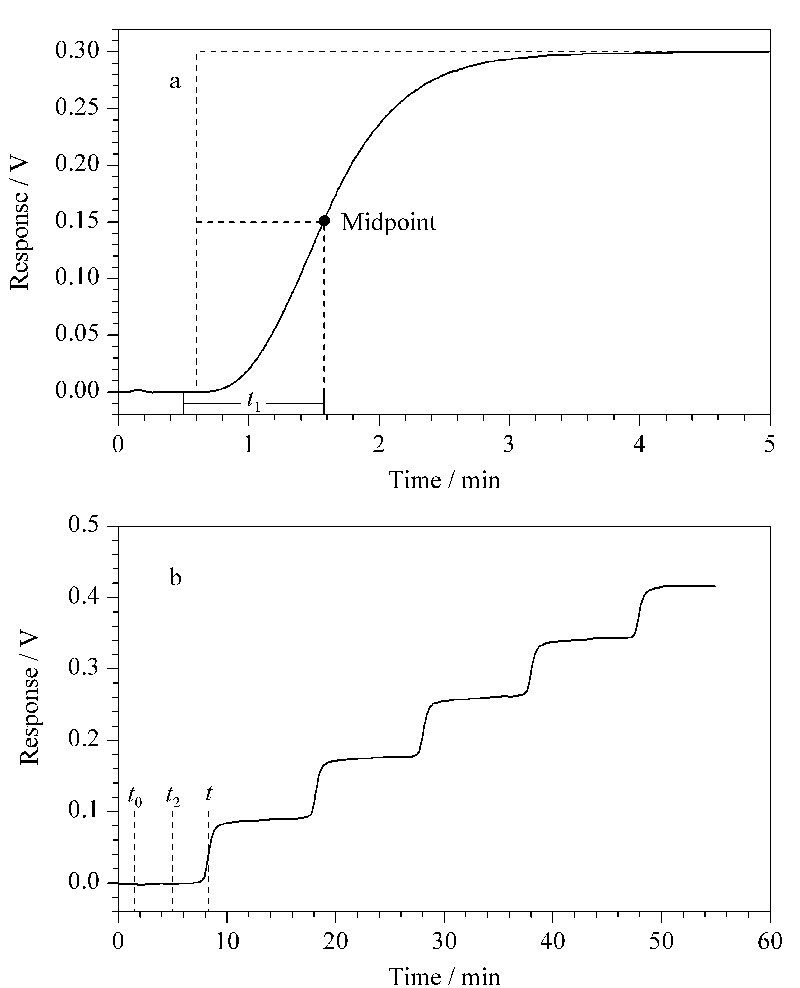
(a)柱外流经时间*t*_1_和(b)吸附量增量Δ*q*的计算方法图

吸附量计算方法 采用中点法计算吸附量,见图3b和公式(1)。


(1) Δ*q=*(*t-t*_0_*-t*_2_)*v*Δ*c/V*_a_

其中Δ*q*为吸附增量;*t*是对应浓度下的样品突破时间,即读取每个浓度下曲线上升到一半高度时对应的保留时间;*t*_1_是柱外流经时间(见图3a);*t*_2_表示对应浓度转换的时间点,由设定的梯度条件决定,如本实验中为10 min;*v*为流动相流速(量);Δ*c*为每一阶梯样品浓度增量;*V*_a_表示色谱柱中固定相的体积。

1.2.4 环孢素A的纯化

称取环孢素A粗品适量用甲醇溶解,配制质量浓度为500 g/L的样品溶液,过0.22 μm膜。C18色谱柱(150 mm×4.6 mm, 10 nm, 10 μm);流动相组成:A为水,B为乙腈,梯度洗脱:0~60 min, 65%B~75%B; 60~80 min, 75%B;流速:0.6 mL/min;柱温:55 ℃;检测波长:203 nm,进样量:30 μL;按照时间对色谱峰进行收集,每2 min收集一次。

馏分纯度分析:Unitary C18色谱柱(250 mm×4.6 mm, 5 μm, 10 nm, 浙江华谱新创科技有限公司),流动相:乙腈-水-磷酸-甲基叔丁基醚(490/460/1/50, v/v/v/v),等度洗脱,流速1.5 mL/min,柱温60 ℃。

## 2 结果与讨论

### 2.1 流动相种类与比例对环孢素A保留的影响

甲醇和乙腈是RPLC中两种常用的洗脱剂。在C18固定相上,分别考察CsA在甲醇-水和乙腈-水两种体系下的保留情况,流动相组成与保留因子(*k*)的关系见图4。

**图 4:**
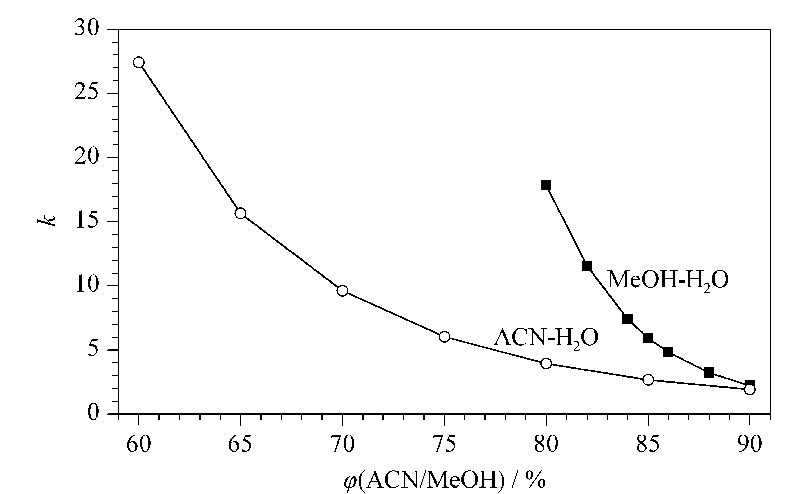
环孢素A在甲醇-水和乙腈-水流动相体系中的保留因子

在甲醇-水体系中,随着甲醇比例降低,CsA的保留时间明显增加。当甲醇-水的体积比为90/10时,*k*≈2,当调整为80/20时,*k*增加到18。对比84%、86%、88%甲醇的保留,甲醇每增加2%, *k*会增加3左右。控制甲醇比例在84%~88%, CsA的*k*在3~7范围内,保留适中。

在乙腈-水体系中,当乙腈比例由90%降低至65%, CsA的*k*由1增加至17左右。在乙腈比例改变5%的情况下(75%、80%、85%), CsA的*k*相差3左右,控制乙腈比例在75%~85%, *k*在3~6范围内。通过有机相比例对保留影响的考察,注意到CsA的保留对于有机相比例改变较为敏感,在有机相比例稍有变动时,保留相差较大,这就需要注意提高有机相比例控制的精度,才能保证分离的稳定性。相对来讲,乙腈比例改变对于CsA保留的影响不及甲醇那样明显。

### 2.2 上样量对环孢素A峰形的影响

根据制备色谱的非线性理论,峰形是影响制备纯度的关键因素。实验分别考察了两种流动相体系下CsA的峰形与上样量之间的关系见图5。无论是甲醇-水体系(图5a1~a3),还是乙腈-水体系(图5b1~b3),随着上样量的增加,CsA的峰形都由对称开始变得拖尾。如果溶质保留时间长,拖尾现象尤其明显,保留时间前移。此实验结果提示,在进行CsA纯化时,需要注意保留时间在目标物前的杂质(即前杂)的分离。根据制备色谱的非线性理论,随着上样量的增加,前杂会因为挤压效应而影响主峰分离的纯度。另外,两种常用的有机相,无论是质子供体的甲醇,还是质子受体的乙腈,在所测试的浓度范围内,在保留相当的情况下,峰形和展宽程度是相似的,即无法通过改变有机相种类对峰形和展宽程度进行调节。

**图 5:**
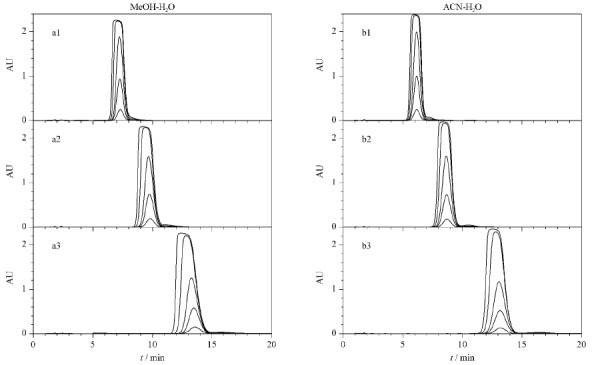
甲醇-水及乙腈-水流动相体系中环孢素A的峰形随上样量的变化

### 2.3 环孢素A的吸附等温线

采用连续前沿吸附法,分别测定环孢素A在6种条件下的吸附等温线(见图6),其中,横坐标表示流动相中溶质的质量浓度*C*(g/L),纵坐标表示溶质在固定相上的吸附量*q*(g/L)。在3种不同比例甲醇-水或者乙腈-水体系中,当流动相中CsA浓度较低时,有机相比例对样品在固定相上的吸附量影响并不明显,而当流动相中溶质质量浓度继续增加到0.5 g/L以上时,如果流动相中有机相比例降低,CsA在固定相上的吸附量则逐渐增加。当流动相中的样品质量浓度达到5 g/L时,在88%甲醇中,吸附量为24.9 g/L,而在84%甲醇中,吸附量增加至40.8 g/L(见图6a)。在乙腈-水体系中,CsA在固定相上的吸附量比在甲醇-水体系中更大些。同样在流动相中样品质量浓度在5 g/L时,在75%乙腈中,吸附量增加至46.4 g/L(见图6b)。当采用制备色谱进行纯化时,采用乙腈-水体系,对于增加CsA在固定相上的吸附量是更有利的。虽然有机相的比例降低有利于改善分离和提高在固定相上的吸附量,但是会增加制备时间和导致峰展宽,所以有机相比例的选择要兼顾分离度、吸附量、分离时间和峰展宽众多因素。

**图 6:**
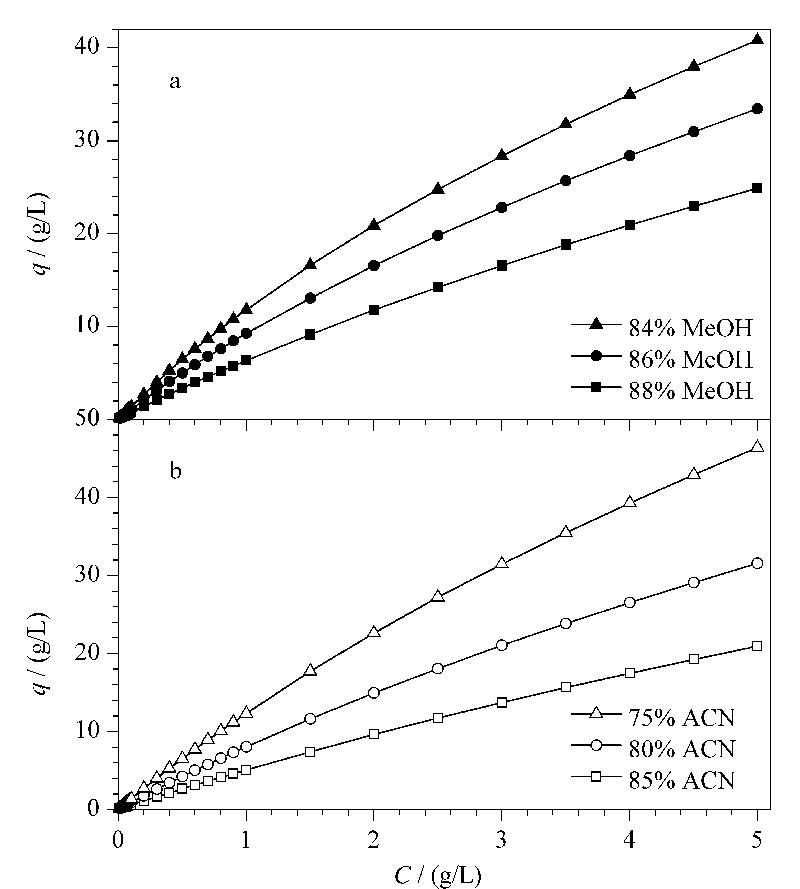
环孢素A在(a)甲醇-水和(b)乙腈-水中的吸附等温线

用*q/C*对*q*作图,在甲醇-水和乙腈-水体系下得到了近似的结果(见图7)。*q/C*随着*q*增加而递减,意味着吸附等温曲线的斜率逐渐减小,符合Langmuir型吸附行为,对应Langmuir峰形(即拖尾峰),与2.2节峰形考察结果一致。当流动相中样品的质量浓度在0.01~0.03 g/L时,*q*/*C*的值随着吸附量*q*的增加迅速下降,此时CsA的峰形会随着上样量的增加而拖尾。而在低有机相比例中(84%甲醇或75%乙腈),这种趋势会减弱。

**图 7:**
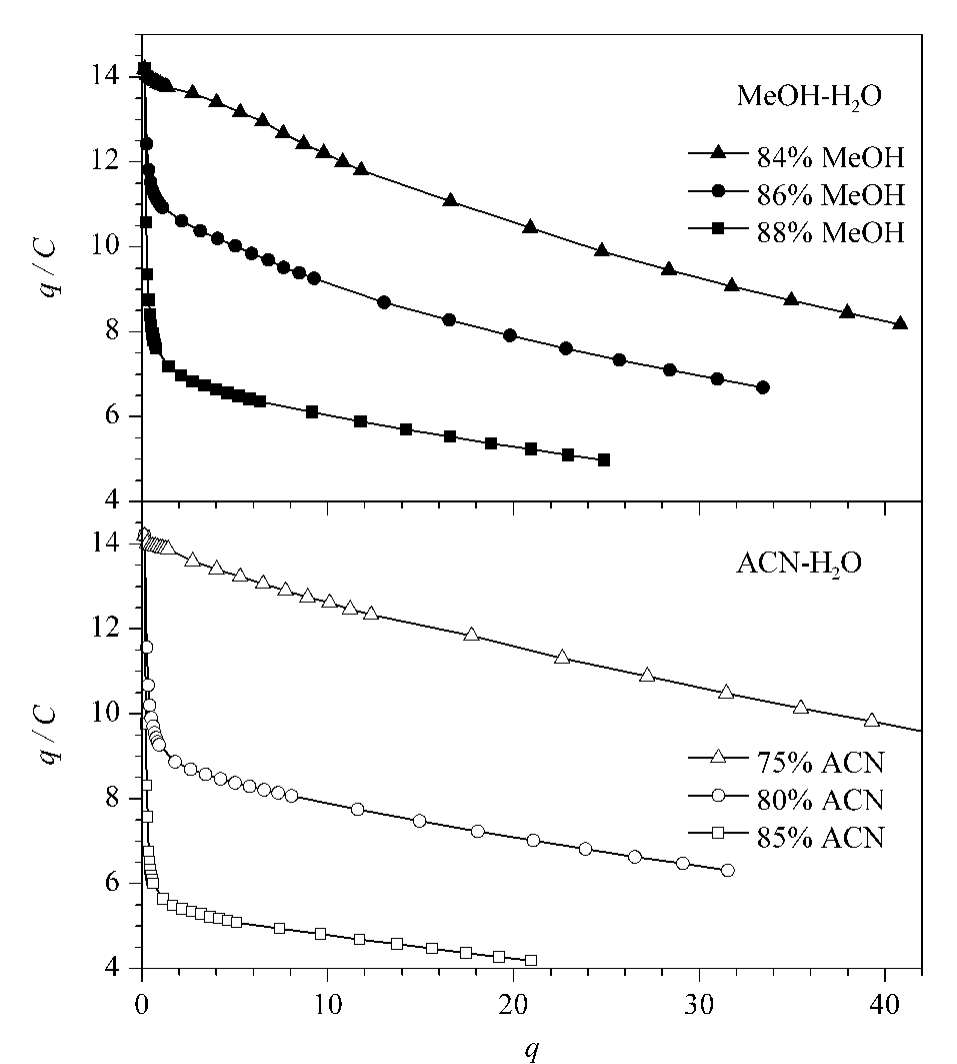
环孢素A在甲醇-水和乙腈-水流动相体系下的Scatchard分析图

用吸附等温线模型对以上数据进行拟合,在甲醇-水体系中,最佳模型为Langmuir模型^[18]^,即


(2) $q=q^{*} \frac{b C}{1+b C}$

而在乙腈-水体系中,最佳模型为Moreau模型^[19]^,即


(3) $q=q^{*} \frac{b C+I(b C)^{2}}{1+2 b C+I(b C)^{2}}$

其中q^*^为单层吸附饱和量,*b*为吸附位点的吸附平衡常数,*C*为流动相中样品的质量浓度,*I*为溶质间相互作用力。

此拟合结果说明,CsA在甲醇-水体系中,固定相与溶质之间为单层吸附,只有一类吸附位点起主要作用。随着甲醇比例降低,单层饱和吸附量q^*^增大,而吸附平衡常数*b*减小,样品之间的相互作用力可以忽略(见表1)。Moreau模型是Langmuir模型的一个简单延伸,因此固定相与CsA之间仍为单层吸附,但是溶质之间的相互作用力是不能忽略的。推测这种差异主要是由于有机溶剂类型不同造成的。在甲醇-水体系中,甲醇是质子型溶剂,会对CsA分子之间的作用力,比如氢键作用产生一定的破坏作用,而乙腈是非质子型溶剂,对这种分子间作用力破坏能力弱,这也说明了乙腈浓度越高,溶质之间的分子作用力越强。如表1所示,随着乙腈比例从85%降低至75%,饱和吸附量q^*^由123 g/L增加到197 g/L,而*I*由0.618降低到0.588。对比两种体系下的参数,在乙腈-水体系下,固定相对溶质有更大的吸附容量。

**表 1:** 甲醇-水和乙腈-水中环孢素A的吸附模型参数

Mobile phase	Volume ratio	q^*^	b	I	R^2^
MeOH-H_2_O	84/16	104.335	0.127		0.9998
	86/14	95.778	0.156		0.9998
	88/12	90.936	0.075		0.9999
ACN-H_2_O	75/25	197.104	0.068	0.588	0.9999
	80/20	154.067	0.056	0.603	0.9999
	85/15	123.012	0.044	0.618	0.9999

q^*^: saturation capacity of the monolayer adsorption; *b*: adsorption equilibrium constant; *I*: interaction between the adsorbates; *R*^2^: coefficient of determination.

### 2.4 环孢素A的纯化制备探索

根据以上研究结果选择CsA的分离体系。从CsA的保留来看,甲醇-水或者乙腈-水通过调整比例都可以获得合适的保留,而且两者在上样量增加时,峰形变化是相似的。相对来讲,乙腈的比例变化对保留影响不那么剧烈,有利于分离稳定性的控制,而且在乙腈-水条件下,CsA在固定相上的吸附量更大。在乙腈-水条件下优化了方法,使杂质有了更好的分离。实验也考虑采用小浓度范围的线性梯度的洗脱方式,一方面有利抑制峰展宽,而且也有利于保持分离的稳定。综上所述,尝试采用0~60 min 65%~75%ACN, 60~80 min 75%ACN的条件对环孢素A进行分离(见图8)。上样量为15 mg,按照时间对色谱峰进行收集和纯度分析,合并保留时间为28~42 min的馏分,可以将主峰的色谱纯度由粗品的89%提高到99.8%,控制杂质含量小于0.2%,回收率为77.7%。此方法可以作为反相色谱模式下纯化CsA的参考。

**图 8:**
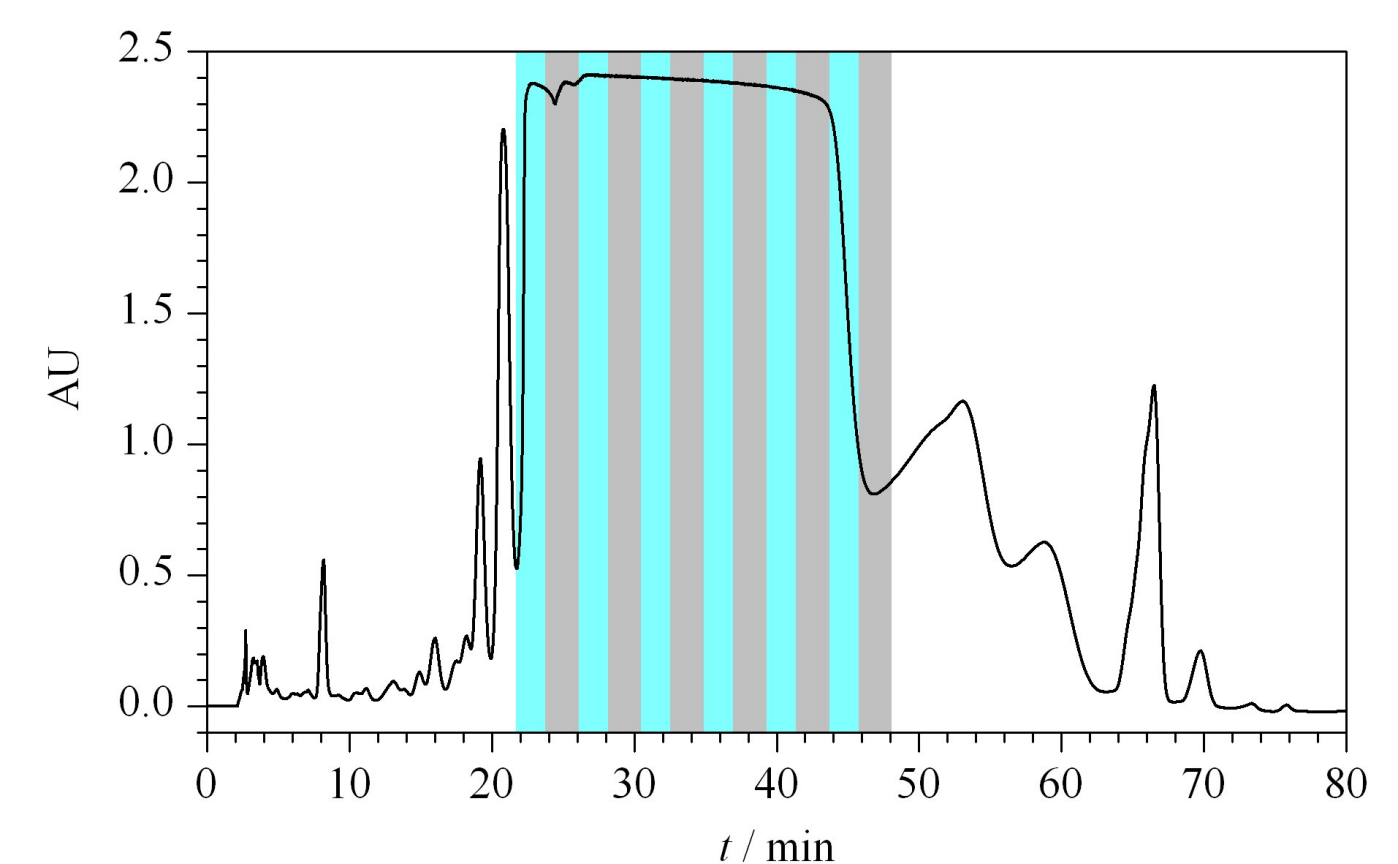
环孢素A的制备分离色谱图

## 3 结论

本文开展了反相色谱模式下CsA保留行为的研究工作,考察了影响CsA峰形的关键因素,通过测定吸附等温线深入认识CsA在反相色谱中的吸附行为。研究结果为CsA进一步的制备纯化提供了理论依据。和已有的正相色谱方法相比,本工作发展的高效反相色谱制备方法流动相条件简单,在纯化效率和自动化控制方面具有一定的优势。后续工作将充分考察本方法的实际工业应用价值。
